# Assessing the Optimal Antibacterial Action of *Lavandula stoechas* L., *Thymus zygis* L., and *Eucalyptus camaldulensis* Dehnh Essential Oils

**DOI:** 10.3390/life14111424

**Published:** 2024-11-05

**Authors:** Farah Aabouch, Saoussan Annemer, Badr Satrani, Ismail Ettaleb, Mohammed Kara, Mohamed Ghanmi, Abdelaaty Abdelaziz Shahat, Ravish Choudhary, Abdellah Farah, Mohamed Ouajdi, Jamila Dahmani

**Affiliations:** 1 Plant, Animal Productions and Agro-Industry Laboratory, Faculty of Science, Ibn-Tofail University, BP 133, Kenitra 14000, Morocco; farahaabouch@gmail.com (F.A.); jamdahmani@gmail.com (J.D.); 2 Laboratory of Microbiology and Chemistry of Aromatic and Medicinal Plants, Forest Research Center, BP 763, Agdal, Rabat 10050, Morocco; badrsat@yahoo.fr (B.S.); ettaleb1ismail@gmail.com (I.E.); ouajdim@gmail.com (M.O.); 3 Laboratory of Applied Organic Chemistry, Faculty of Sciences and Techniques, University Sidi Mohammed Ben Abdellah, BP 2202, Fes 30000, Morocco; saoussan.annemer@usmba.ac.ma (S.A.); farah.abdellah1@gmail.com (A.F.); 4 Botany, Mycology and Environment Laboratory, Department of Biology, Faculty of Sciences, Mohammed V University, Rabat 10050, Morocco; 5 Laboratory of Biotechnology, Conservation and Valorisation of Natural Resources (LBCVNR), Faculty of Sciences Dhar El Mehraz, Sidi Mohamed Ben Abdallah University, BP 1796, Atlas, Fes 30000, Morocco; 6 National Office for Agricultural Consultation, BP 6672, Rabat 10050, Morocco; ghanmi@gmail.com; 7 Pharmacognosy Department, College of Pharmacy, King Saud University, Riyadh 11451, Saudi Arabia; ashahat@ksu.edu.sa; 8 Division of Seed Science and Technology, ICAR-Indian Agricultural Research Institute, New Delhi 110012, India; ravianu1110@gmail.com

**Keywords:** antibacterial activity, essential oil, *Lavandula stoechas*, Thymus zygis, *Eucalyptus camaldulensis*, augmented Simplex centroid

## Abstract

The use of combined essential oils (EOs) is a new technique that can improve their preservative effects while minimizing their sensory impact in foods. The aim of this study was to determine the chemical profile of three essential oils (EOs) extracted from *Lavandula stoechas* L. (Ls), *Thymus zygis* L. (Tz), and *Eucalyptus camaldulensis* Dehnh (Ec) and to evaluate their synergistic antibacterial activity for optimal inhibition against *Bacillus subtilis*, *Escherichia coli,* and *Staphylococcus aureus* using an augmented Simplex centroid mixing scheme. The essential oils were extracted by hydrodistillation and analyzed via gas chromatography–mass spectrometry. Anti-bacterial potency was evaluated by disk diffusion. Chemical analysis revealed the main compounds in *Lavandula stoechas* (Ls) essential oil: camphor (36.15%), followed by fenchone (16.57%) and Z-8-hydroxy linalool (8.28%). The *Thymus zygis* (Tz) essential oil is dominated by δ-terpineol (27.64%), δ-3-carene (15.7%), and thymol (14.17%). In contrast, the *Eucalyptus camaldulensis* (Ec) essential oil contains mainly 1,8-cineole (43.61%), γ-terpinene (11.71%), and α-terpineol (10.58%). The optimal mixture is the binary association of 40% *E. camaldulensis* EO and 60% *T. zygis* EO, which provides an effective inhibition diameter (ID) of 13.37 mm to inhibit *S. aureus*. Furthermore, the formulation of 27% and 73% EOs of *E. camaldulensis* and *T. zygis*, respectively, corresponds to the mixture required to achieve the optimum inhibition diameter (ID = 11.55 mm) against *E. coli*. In addition, the mixture of 29% EO of *E. camaldulensis* and 71% EO of *T. zygis* is the optimum mixture to inhibit *B. subtilis*, with an inhibition diameter of 12.31 mm. These findings highlight the potency of antibacterial formulations of these essential oils and suggest that they might be used as substitutes for conventional drugs to prevent the development of bacteria responsible for serious infections and food spoilage.

## 1. Introduction

For many years, the food industry has relied on synthetic preservatives to prevent microbial contamination in packaged foods [1]. However, these artificial preservatives pose significant health risks, causing side effects such as headaches, nausea, weakness, cancers, and anorexia [2]. Consequently, the food industry is moving towards using natural food products or extracts, which require protection against spoilage and microbial contamination throughout their shelf life.

For thousands of years, medicinal and aromatic plants have been utilized to combat human-infectious diseases due to their preservative and pharmacological properties. Numerous secondary metabolites from these plants have shown significant biological activities, making them highly valuable [3]. Among these, essential oils (EOs) stand out as concentrated hydrophobic liquids rich in volatile plant-derived compounds. They have been employed for various medical and health purposes for millennia [4]. The biological activities of essential oils, including antioxidant, antifungal, and antibacterial effects, are well documented [5,6]. Additionally, their safe application as natural food preservatives have been noted [7]. However, achieving similar antibacterial and preservative effects observed in laboratory tests often requires high concentrations [8], leading to potential overdoses and alterations in food flavor. As a result, research has shifted to exploring the synergistic effects of different essential oil combinations to enhance their efficacy while reducing the necessary concentration [9].

*Lavandula stoechas* L., belonging to the Lamiaceae family, is an evergreen shrub characterized by its foliage and dark-purple-to-violet flowers, which are highly aromatic. This plant can reach a height of up to 100 cm and grows naturally in Mediterranean regions. In Morocco, it is found in various areas, particularly in the Rif, Middle Atlas, and High Atlas regions [10,11]. Used for its medicinal properties, its benefits are attributed to its bioactive compounds, such as camphor, terpineol, eucalyptol, fenchone, and linalool [12,13]. Thanks to its phytochemical composition, *L. stoechas* is widely employed in traditional medicine as well as in the food and cosmetic industries. In Morocco, it is used to treat inflammations, nephrotic syndromes, rheumatic diseases, and as an antispasmodic agent. It is also recognized for its antidiabetic properties and its use in the treatment of hypertension [14]. Research has highlighted its essential oils’ antimicrobial, antioxidant, antileishmanial, insecticidal, anti-inflammatory, and anticancer activities [15,16,17]. These biological properties are attributed to its high fenchone/camphor chemotype content. However, the results of its biological activities vary from study to study, and these differences may be due to variations in the chemical compositions of its essential oils, influenced by environmental conditions and regional differences [18].

*Thymus zygis* L. is an aromatic plant from the Lamiaceae family, is widely distributed in the Iberian Peninsula, and has a long history of use as a spice. It is characterized by its small, linear, lanceolate leaves and produces clusters of small tubular flowers that range in color from white to pale pink. It grows in Mediterranean regions and is found in Morocco, where it develops spontaneously in forest clearings, rocky pastures of low to medium mountains, primarily in the High Atlas, North Atlantic, Middle Atlas, Essaouira region and the Mediterranean coast, particularly in cold, semi-arid, humid, and sub-humid bioclimatic areas [19]. Although this species is widely used for its therapeutic benefits in the pharmaceutical, cosmetic, and perfumery industries [20], the essential oil of *T. zygis* mainly contain phenolic compounds, such as thymol, and alcoholic compounds, such as terpineol, which contribute to their various bioactive properties, including anti-inflammatory, antifungal, antimicrobial, and antioxidant effects [21,22]. While the major components are well-known for their biological significance, the minor constituents also play an important role in enhancing the effects of the main components through synergistic and additive mechanisms [23,24].

*Eucalyptus camaldulensis* Dehnh, native to Australia, is a species that belongs to the Myrtaceae family [25]. In Morocco, it is primarily planted in the northwestern region of the country [26]. Eucalyptus trees are large and can reach heights of over 100 m. Their evergreen, aromatic leaves are entire, leathery, and have a high cutin content [27]. The plant is widely used in traditional therapies for various ailments, particularly as an antiseptic and astringent [28]. Numerous studies have focused on the cosmetic and pharmaceutical applications of *E. camaldulensis* leaves, especially in the treatment of respiratory diseases [29]. Furthermore, many studies have examined the antioxidant, antimicrobial, and antifungal properties of its essential oils [30]. The main compounds typically found in the essential oil of *E. camaldulensis* leaves include 1,8-cineole, γ-terpinene, p-cymene, and α-pinene [31,32]. Notably, 1,8-cineole is considered a major bioactive component due to its numerous biological activities.

This study aimed to evaluate the antibacterial effects of the combined essential oils of three plants, *L. stoechas*, *T. zygis,* and *E. camaldulensis*, against three bacterial strains: *Bacillus subtilis*, *Staphylococcus aureus,* and *Escherichia coli*. This combination was chosen with the aim of enhancing efficacy and reducing the required amount of essential oils, thereby minimizing their toxicity and negative impact. To achieve this, an augmented Simplex centroid mixture design was employed to develop polynomial models to highlight the synergy between the essential oils against the bacterial strains.

## 2. Materials and Methods

### 2.1. Plant Material

Samples of the aerial parts of *T. zygis*, *L. stoechas,* and *E. camaldulensis* were collected in June 2021 from the regions of El Hoceima (North Morocco: 35°08′09.8″ N; 4°05′10.7″ W), Azrou (Middle Atlas of Morocco: 33°25′48″ North, 5°12′36″ West), and Mamora (Northwest Morocco: 34°16′16.0″ N; 6°25′28.1″ W), respectively. The identification of the species was confirmed at the Scientific Institute of Rabat (Morocco) by Mohammed Sghir Taleb (a research professor at the Scientific Institute. His research focuses on botany, plant ecology, aromatics, and socio-economics).

### 2.2. Extraction of Essential Oils

The aerial parts of *L. stoechas*, *T. zygis,* and *E. camaldulensis* were subjected to hydrodistillation using a Clevenger-type apparatus [33]. Three distillations were carried out by boiling 200 g of plant material with two liters of distilled water for three hours. The obtained essential oils were placed in hermetically sealed glass vials and stored at a temperature of 4 °C until use. The essential-oil yield was determined relative to the dry matter and evaluated from 3 samples of 20 g dried for 48 h in an oven at 60 °C. The essential oil yields of the samples were determined using the formula specified by [34]. All experiments were performed in triplicate.

Yield % = (weight of EO obtained by distillation (g)/weight of dry biomass (g)) × 100;

### 2.3. Gas Chromatography–Mass Spectrometry (GC/MS) Analysis

The essential oils were subjected to chemical analysis using gas chromatography coupled with mass spectrometry (GC/MS) and flame ionization detection (GC–FID). The GC/MS analysis was employed to quantify the components, while the GC–FID analysis was used for identification purposes. All samples were analyzed by gas chromatography using an HP-5 capillary column (30 m × 0.25 mm, film thickness 0.25 µm), an FID detector, and an injector set at 275 °C. The equipment was sourced from Hewlett–Packard, located in Palo Alto, California, USA. After an initial five-minute interval, the oven temperaturewas gradually increased from 50 °C to 250 °C at a rate of 4 °C/min. Nitrogen was used as the carrier gas at a flow rate of 1.8 mL/min. The samples, diluted 1/50 in methanol, were injected in a volume of 1 µL in split mode at a ratio of 1/50 and a flow rate of 72.1 mL/min. The proportions of the components present in the essential oils were expressed as percentages, determined by peak area normalization. Retention indices (RI) on the HP-5 MS column were calculated using a homologous series of alkanes ranging from C9 to C28.

The gas chromatography–mass spectrometry (GC/MS) analysis was performed using a Hewlett–Packard gas chromatograph (HP 6890) coupled to a mass spectrometer (HP and stationary syringe 5973). An HP-5MS column (30 m × 0.25 mm, 0.25 µm film thickness) was used. The column temperature was initially set at 50 °C, then gradually increased to 250 °C at a rate of 2 °C/min. Helium gas with a purity of 99.995% served as the carrier gas, flowing at a rate of 1.5 mL/min, with a split ratio of 1/74.7, corresponding to a flow rate of 112 mL/min. Components were identified using a NIST 98 spectral library in the mass spectrometer. The ionization voltage was maintained at 70 eV, the ion source temperature was set at 230 °C, and the mass scan range was between 35 and 450 m/z. Component identification was verified by comparing the elution order of the compounds with the relative retention indices reported in the literature.

### 2.4. Tested Organisms

The bacterial strains *Bacillus subtilis* (ATCC 6633), *Escherichia coli* (ATCC 8739), and *Staphylococcus aureus* (ATCC 6538) belong to the collection of the Microbiology Laboratory at the Center for Innovation, Research, and Training, Rabat, Morocco. The strains were inoculated from a master culture maintained on agar at 4 °C, placed on nutrient agar plates, and incubated at 37 °C for 24 h.

### 2.5. Antibacterial Activity of Essential Oils

The antibacterial activity of the essential oils (EOs) was evaluated using a disk diffusion method, known for its reliability and reproducibility. This method involves placing a sterile disk soaked in EO on a freshly growing bacterial lawn and measuring the inhibition zone diameter, which reflects the antibacterial activity of the EOs. For this test, 15 mL of Tryptic Soy Agar (TSA) was poured into each Petri dish, and 100 µL of a bacterial suspension with a density equivalent to 0.5 McFarland standard (10^8^ CFU/mL) was added. Sterile filter paper disks (6 mm) were then impregnated with 5 μL of EO and placed on the inoculated Petri dishes. Tetracycline (300 µg) was used as a positive reference standard to determine the sensitivity of the tested strains. The Petri dishes were incubated at 37 °C for 24 h. After incubation, the diameter of the inhibition zone was measured in millimeters. All tests were conducted in triplicate [35].

### 2.6. Mixture Design and Statistical Analysis

The mixture design is an experiment in which the responses are presumed to be influenced by the relative proportions of the mixture’s constituents, rather than the total quantity of the mixture. This approach was implemented to identify the optimal formulation while simultaneously reducing the number of experiments. Consequently, it enables the identification of the correlation between the variables and the experimental responses that were quantified.

#### 2.6.1. Chosen Design

**Figure 1:**
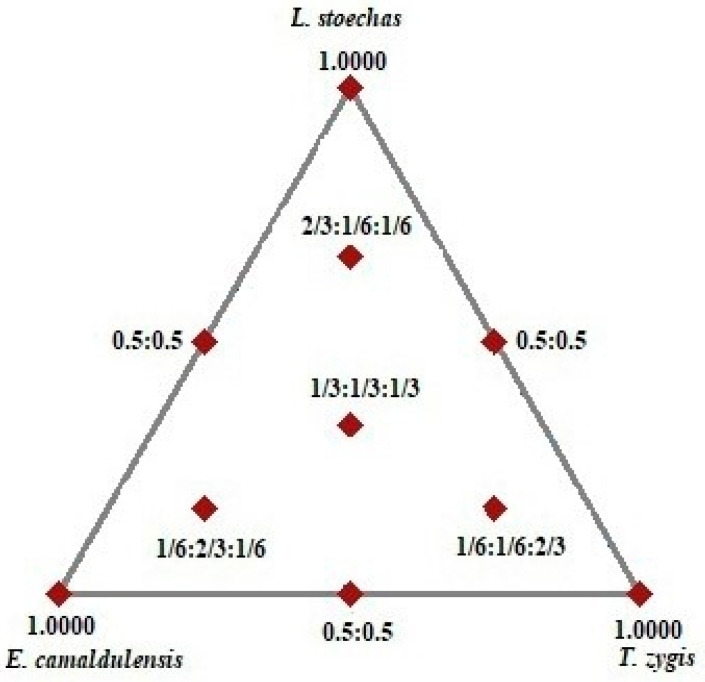
Positions of experimental points for augmented Simplex-centroid designs.

In this work, an augmented centroid design (Figure 1) was employed to determine the optimal formulation components that provide the most effective combination of EOs to achieve increased antibacterial activities (i.e., the highest diameter of inhibition (DI)).

To establish the experimental setup, a polynomial model explaining the relationship between a response and the factors under consideration was constructed using the experimental data of the selected design. This design includes ten experiments divided as follows: three diluted EOs in the triangle’s vertices (experiments 1, 2, and 3), 0.5/0.5 mixtures (experiments 4, 5, and 6), an equal proportionate mixture of the three constituents (experiments 7), and control points (experiments 8, 9, and 10) [36]. To evaluate pure error and compare it with lack of fit, experiment 7 was repeated three times, resulting in 12 experiments for this design. The sum of the components of the mixture was 100%.

$$\sum_{i=1}^{n}x_{i\;}=100\%.$$

Table 1 shows 12 ternary combinations of the three Eos (*L. stoechas*, *E. camaldulensis*, *and T. zygis*) synthesized using an augmented centroid design.

#### 2.6.2. Chosen Mathematical Model

The chosen model is a special cubic model, which is a linear model with third-order interactions, having the following general form:

$$Y=\alpha_{1}X_{1}+{\;\alpha }_{2}X_{2}+{\;\alpha }_{3}X_{3}+{\;\alpha }_{12}X_{1}X_{2}+{\;\alpha }_{13}X_{1}X_{3}+{\;\alpha }_{23}X_{2}X_{3}+\alpha_{123}X_{1}X_{2}X_{3}+ɛ$$

With:

- Y represents the response expressed in mm for the diameter of inhibition (DI) and in µg/mL for IC50.
- α1, α2, α3 are the coefficients of the linear terms.
- α12, α23, α13 are the coefficients of the binary interaction terms.
- α123: coefficient of the ternary interaction term.
- ɛ: error term.

#### 2.6.3. Statistical Analysis

To evaluate the significance of the fitted model, an ANOVA test was performed. The F_ratio (CMR/CMr)_, which is the ratio of the mean square due to regression to the mean square due to residuals, was calculated. To assess the quality of the model fit, we also calculated the F_ratio (CMR/CMr)_, which is the ratio of the mean square due to lack of fit to the mean square due to pure error, as described by [37].

The quality of the chosen model was expressed by the coefficient of determination, *R*^2^. A value closer to 1 indicates that the variability explained by the model is much greater than the variability explained by the residuals [38].

To determine the significance of factors, a *t*-test was employed at a significance level of 95%. This test involves calculating the ratio of the coefficient value to its standard error. The resulting statistic is the *t*-value, from which the probability that the coefficient is zero can be assessed. In the coefficient table, each factor is accompanied by its *t*-value and *p*-value. A smaller *p*-value indicates greater statistical significance of the coefficient [37].

To finalize the optimization step, the mixture design was used to identify a compromise setting that leads to the desired response. Additionally, the “Desirability” function was applied to determine the precise optimal setting with a compromise percentage [37].

## 3. Result and Discussion

### 3.1. Essential Oil Yields

**Figure 2:**
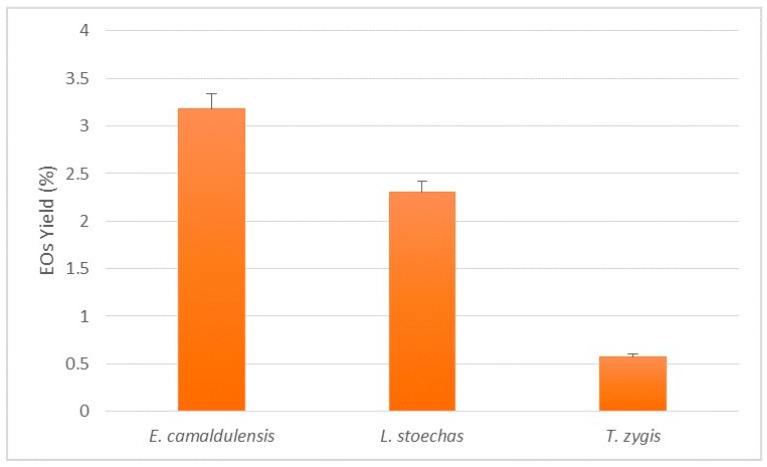
Average yield of the essential oils as a function the plants studied.

The hydrodistillation extraction of essential oils from *E. camaldulensis*, *L. stoechas*, and *T. zygis* yielded 3.18%, 2.30%, and 0.57%, respectively (Figure 2). Different results have been reported for *E. camaldulensis* essential oils in previous studies: in Iran, the yield was 2.10% [39], in Malaysia, it ranged from 0.46% to 1.4% [40], while in Nicosia, Cyprus, it was reported to be approximately 2.40% [41]. Similarly, the yield of *L. stoechas* essential oils varies across different regions: in Sidi Slimane, Northwest Morocco, it was 2.10% [42], in Tunisia, it ranged from 0.72% to 0.95% [43], and in Italy, it was notably lower, at 0.11% [44]. For *T. zygis* from Taza, Northeast Morocco, a low yield of 0.3% was reported [45], whereas *T. zygis* subsp. *sylvestris* from Portugal showed a higher yield of 1.2% [46].

### 3.2. Chemical Composition of Essential Oils

The main components present in the essential oils obtained by hydrodistillation and the blend of essential oils according to the mixture design plan are summarized in Table 2. Chromatographic analyses by GC and GC/MS identified 60 compounds in *L. stoechas* essential oil, accounting for 99.49% of the composition. Monoterpenes were the predominant components in *L. stoechas* essential oil, comprising 84.15%, followed by sesquiterpenes at 15.24% (Table 2). The predominant compounds were camphor (36.15%), fenchone (16.57%), and Z-8-hydroxy linalool (8.28%). Comparisons with the chemical composition of *L. stoechas* essential oils from other countries revealed qualitative and quantitative differences. For instance, in Algeria, α-fenchone (39%), camphor (18.5%), and bornyl acetate (7.79%) were dominant [47]. In Tunisia, camphor (15.32–50.63%), fenchone (6.57–34.70%), and 1,8-cineole (0.05–13.45%) were found to be major constituents [11]. In Taounate, Northern Morocco, camphor (43.97%), fenchone (30.39%), and camphene (4.09%) dominated [48].

*T. zygis* essential oils revealed the presence of 54 compounds constituting 99.63% of the total composition. The major components were δ-terpineol (27.64%), δ-3-carene (15.7%), thymol (14.17%), dehydro-linalool (4.99%), trans-carvone oxide (4.13%), and α-pinene (3.98%) (Table 2). Monoterpenes represented the dominant class in *T. zygis* essential oil (94.18%), while sesquiterpenes were detected in smaller quantities (5.47%). These results differ from previous studies; for instance, *T. zygis* from Ifrane-Boulemane province, Middle Atlas, Morocco, showed dominance of thymol (36.4%), carvacrol (24.1%), and p-cymene (23.5%) [49]. In Serbia, *T. zygis* essential oil was rich in thymol (35%) and p-cymene (24.1%) [50]. Different distillation techniques revealed that *T. zygis* essential oil from Portugal was dominated by ρ-cymene (10.5–77%), thymol (1.42–41%), linalool (2.43–11.7%), γ-terpinene (1.11–10%) and carvacrol (0.48–8.67%) [51].

Furthermore, *E. camaldulensis* essential oil contained 55 identified constituents, comprising 99.68% of the total compounds. Monoterpenes dominated (90.57%), while sesquiterpenes were present in lower amounts (9.03%). The major components were 1,8-cineole (43.61%), γ-terpinene (11.71%), α-terpineol (10.58%), p-cymene (4.93%), terpinene-4-ol (3.91%), and α-thujene (3.49%) (Table 2). Our findings are consistent with previous reports; for instance, *E. camaldulensis* from Burkina Faso was dominated by 1,8-cineole (44.17%), followed by α-pinene (14.70%) and o-cymene (14.11%) [52]. In Pakistan, *E. camaldulensis* essential oil was rich in eucalyptol (30.43%), α-pinene (10.35%), and spathulenol (10.15%) [53], while in Iran, ρ-cymene (18.86%), α-pinene (16.56%), alloaromadendrene (12.26%), and 1,8-cineole (11.79%) were major components [54].

These variations can be attributed to various factors including environmental conditions such as soil type, precipitation, climate, seasonal conditions, harvest timing, extraction and processing methods, duration of action, plant origin, phenological stage of the plant, and genetic influences [55,56].

Regarding the composition of binary blends of essential oils, the *Ls/Tz* (0.5:0.5) sample contains a significant proportion of monoterpenes (89.15%) and a low proportion of sesquiterpenes (10.25%). The main components are camphor (23.77%), δ-terpineol (16.8%), δ-3-carene (8.87%), trans-oxide linalool (7.67%), thymol (5.91%), trans-oxide carvone (3.95%), and Z-8-hydroxy linalool (3.83%), while fenchone is present in a lower quantity compared to the individual Ls essential oil.

The percentages of 1,8-cineole are similar in both combinations of EO blends *Ls/Ec* and *Tz/Ec* (24.53–21.93%, respectively). However, the percentage of δ-3-carene and δ-terpineol in the *Ls/Ec* sample is less than 10%, reaching 11.31% and 16.15%, respectively, in the *Tz/Ec* sample. In contrast, camphor is present at 23.6% and absent in both combinations *Ls/Ec* and *Tz/Ec*, respectively. The binary EO blends *Ls/Ec* and *Tz/Ec* are characterized by a substantial amount of monoterpenes (87.94%, 86.96%), while there is a low amount of sesquiterpenes (11.57%, 9.35%), respectively.

The tertiary combination *Ls/Tz/Ec* (1/3:1/3:1/3) primarily contains camphor (15.82%), 1,8-cineole (14.32%), and δ-terpineol (11.29%). Other constituents such as δ-3-carene (8.74%), γ-terpinene (4.08%), fenchone (1.28%), α-terpineol (1.98%), and thymol (4.43%) were detected in smaller quantities, although they are present in the individual oils at relatively higher concentrations (over 10%). The percentages of δ-3-carene, fenchone, and thymol conform to the theoretical binary values of the combined EOs *Ls*, *Tz,* and *Ec* in the ratio (1/3:1/3:1/3). Monoterpenes (89.25%) were found at higher levels than sesquiterpenes (10.79%) in the tertiary combination.

The main components detected in the samples, *Ls/Tz/Ec* (2/3:1/6:1/6), (1/6:2/3:1/6), (1/6:1/6:2/3), are similar to those present in the individual essential oils. However, the results showed that the estimated component percentages in these samples are higher compared to the theoretical binary values, where each sample should contain 66.67% of the plant component with a higher ratio in the blend (2/3 ratio). They are dominated by monoterpenes (87.5%, 87.46%, 88.27%), while the quantities of sesquiterpenes are lower (11.86%, 11.5%, 6.07%), respectively.

### 3.3. Simple Antibacterial Activity

The results of the antibacterial activity of the EOs from *L. stoechas*, *T. zygis,* and *E. camaldulensis*, assessed using the disk diffusion method against *Staphylococcus aureus*, *Bacillus subtilis,* and *Escherichia coli*, are summarized in Table 3.

The inhibition zone diameters vary based on the EOs’ nature and the tested species’ sensitivity. *L. stoechas*, *T. zygis,* and *E. camaldulensis* exhibited significant antibacterial effects against *S. aureus*, with inhibition zone diameters of 20.78 ± 0.74 mm, 18.33 ± 0.89 mm, and 25.00 ± 0.22 mm, respectively. This notable antibacterial effect was also observed against *B. subtilis*, with inhibition zones of 20.33 ± 0.44 mm, 17.11 ± 0.30 mm, and 22.56 ± 0.59 mm for *L. stoechas*, *T. zygis,* and *E. camaldulensis*, respectively. Additionally, *E. coli* showed significant sensitivity to the EOs of *L. stoechas*, *T. zygis,* and *E. camaldulensis*, with inhibition zone diameters of 15.78 ± 0.59 mm, 16.11 ± 0.96 mm, and 19.11 ± 0.30 mm, respectively. All tested strains exhibited very strong sensitivity to the antibiotic tetracycline.

Previous studies have reported the antibacterial effects of the three EOs studied. Our results corroborate those reported by [57], which show that the EO of *L. stoechas* causes significant inhibition against the Gram-positive bacterium *Staphylococcus aureus* (18 ± 0.1 mm), while a weak antibacterial effect was noted against the Gram-negative bacterium *Escherichia coli* (12 ± 0.2 mm). Conversely, Ez Zoubi et al. [58] reported a strong antibacterial effect of *L. stoechas* EO against *Staphylococcus aureus* (17.5 ± 3.1 mm) and a significant effect against *Escherichia coli* (14.3 ± 0.55 mm). Additionally, the study reported by [42] showed high antibacterial activity of this species collected from Sidi Slimane (northwest Morocco) against *Bacillus subtilis* (24 mm) and *Escherichia coli* (28.5 mm).

However, it has previously been demonstrated that the EO of *T. zygis* from Serbia exhibits weak antibacterial effects against *Staphylococcus aureus* (5.67 ± 0.58 mm) and *Listeria monocytogenes* (9.00 ± 1.00 mm) and against *Escherichia coli* (7.33 ± 0.58 mm), *Micrococcus luteus* (6.67 ± 0.58 mm), *Pseudomonas putida* (4.67 ± 0.58 mm), and *Enterobacter aerogenes* (12.33 ± 0.33 mm) [59]. However, Coimbra et al. [23] reported that the EO of *T. zygis* from Portugal has a strong antibacterial effect against *Staphylococcus aureus* (20.67–35.10 mm).

The inhibition zone of *E. camaldulensis* EO from Pakistan against *E. coli* ranged from 14.46 ± 0.03 mm to 19.34 ± 0.05 mm, while against *B. subtilis*, it ranged from 10.77 ± 0.05 mm to 15.81 ± 0.04 mm [60].

The antibacterial activity of essential oils generally depends on their chemical composition and the interaction between their functional groups and the bacterial cell wall, as well as the presence of inactive components and their synergistic interactions. The hydrophilic functional groups in essential oils are crucial for their antimicrobial properties. Phenolic compounds are the most effective, followed by aldehydes, ketones, alcohols, ethers, and hydrocarbons [61].

Gram-negative bacteria exhibit increased resistance due to their cell wall structure, which limits the penetration of hydrophobic compounds like essential oils and their bioactive components through the lipopolysaccharide layer. In contrast, Gram-positive bacteria lack this outer membrane and have a cell wall primarily composed of a thick peptidoglycan layer, which facilitates the diffusion of essential oils through their cell membrane [62].

### 3.4. Mixture Design Formulation: Antibacterial Activities

#### 3.4.1. Experimental Design

The different combinations of the three essential oils (EOs) studied and the observed responses for each experiment are detailed in Table 4. The experiments were conducted following randomization, and each response is the average of three repetitions.

According to Table 4, we can clearly observe that the binary mixture of *E. camaldulensis/T. zygis* essential oils (tests containing 0.5:0.5) and the ternary mixture of *L. stoechas/E. camaldulensis/T. zygis* essential oils (tests containing 1/6:1/6:2/3) showed higher inhibition diameters than those of each of the two essential oils separately.

#### 3.4.2. Statistical Validation of the Postulated Model

Based on the analysis of variance table (Table 5), we can conclude that the main effect of the regression is significant, as the *p*-value is less than 0.05. Furthermore, the models do not exhibit a lack of fit since the *p*-value for the lack of fit is greater than 0.05.

**Figure 3:**
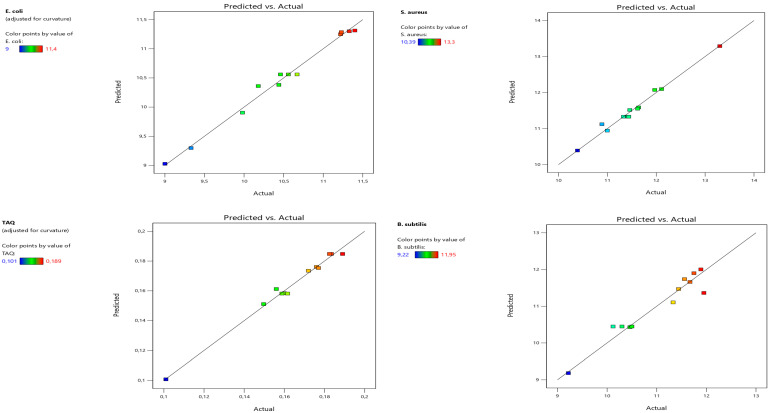
Scatter plot of observed values vs. predicted values.

The coefficients of determination range from 0.9278 to 0.9881, indicating a strong agreement between the experimental and predicted values of the fitted model. These results, confirmed by the graph (Figure 3), demonstrate that the curves of the observed values versus the predicted values closely resemble a straight line.

#### 3.4.3. Effects of Factors and Models

The effects of all studied factors, along with the statistical t-student values and observed probability (*p*-value), are summarized in Table 6.

For *S. aureus*, the statistically significant coefficients are the linear terms b1, b2, and b3. These results indicate that the antibacterial activity against *S. aureus* depends on all terms of the adapted mathematical model, except for the coefficient corresponding to the binary term b13. The chosen mathematical model is represented by the following Equation:

Y = 10.94X_1_ + 10.39X_2_ + 12.10X_3_ + 3.67X_1_X_2_ + 8.18X_2_X_3_ − 30.90X_1_X_2_X_3_ + ɛ

For *E. coli*, the statistically significant coefficients are b1, b2, b3, b12, b13, and b23. These results suggest that the antibacterial effect against *E. coli* is linked to both individual and binary effects. However, no ternary interaction impacts the observed antibacterial action. The chosen mathematical model for the response against *E. coli* is represented by the following Equation:

Y = 10.38X_1_ + 9.03X_2_ + 11.25X_3_ − 1.63X_1_X_2_ + 1.94X_1_X_3_ + 4.59X_2_X_3_ + ɛ

For *B. subtilis*, the coefficients are statistically significant, with *p*-values less than 0.05, except for the coefficient corresponding to the binary term b13, which should be excluded from the proposed model. The most significant terms are those representing the effects of the individual components (b1, b2, b3). The chosen mathematical model is represented by the following Equation:

Y = 11.47X_1_ + 9.18X_2_ + 11.90X_3_ + 5.34X_1_X_2_ + 5.84X_2_X_3_ − 45.09X_1_X_2_X_3_ + ɛ

#### 3.4.4. Optimization of Formulation: Inhibition Zone Response

##### Mixing Profile

The objective of this Section is to identify the optimal formulation of the three EOs that result in an inhibition zone diameter, indicating a sensitivity classified as ‘extremely sensitive’ [63]. Therefore, we will search for the proportions of the components that provide a maximum value of inhibition zone diameter.

**Figure 4:**
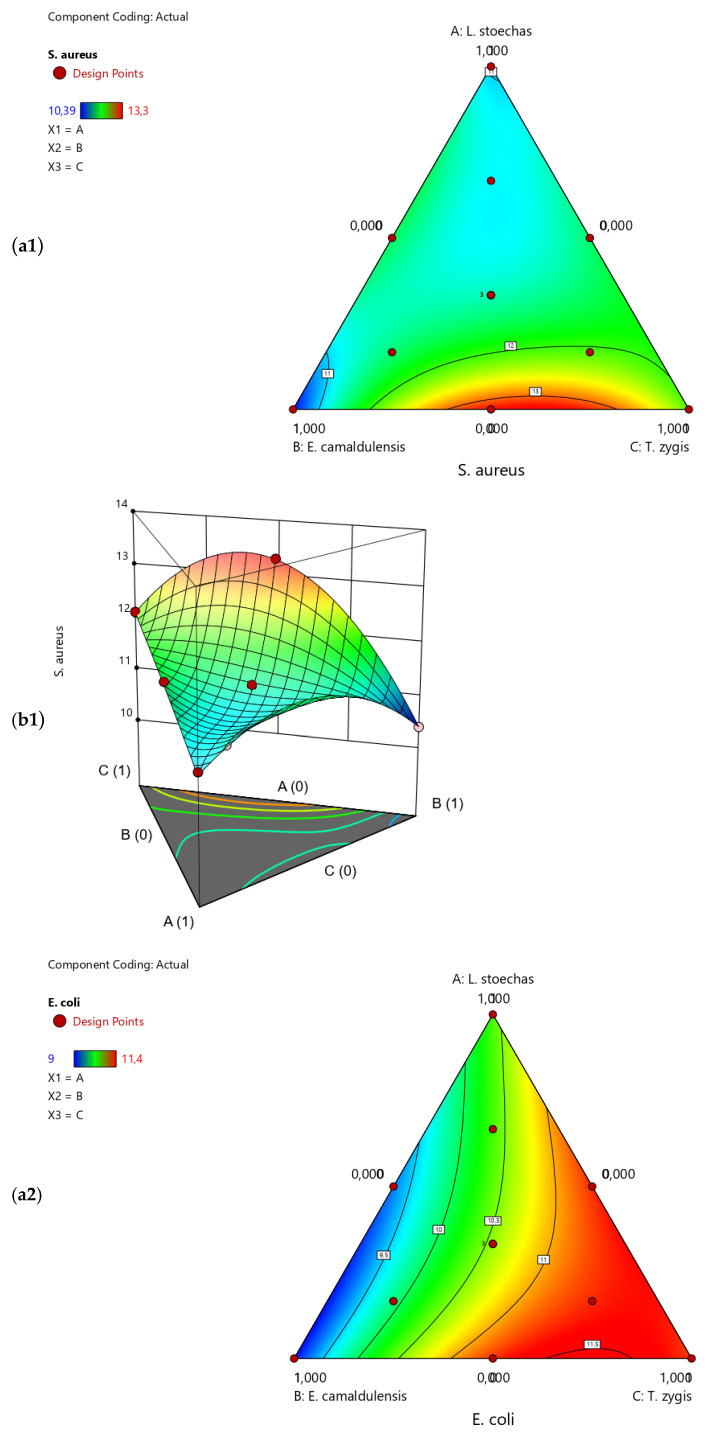

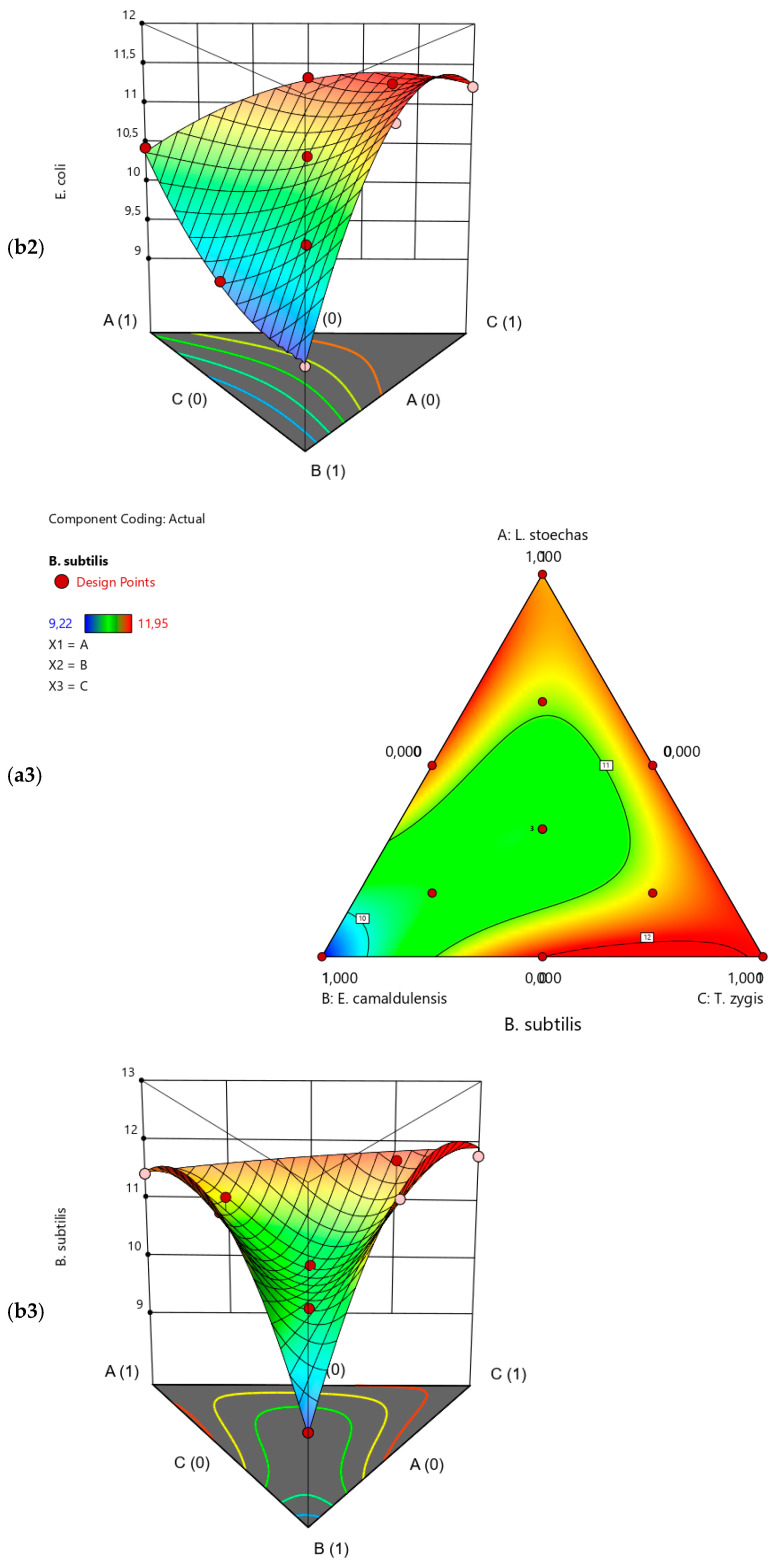
(**a**) Two-dimensional mixing profile and (**b**) three-dimensional profile showing the zone of maximum di response based on the three constituents.

The mixture plot (Figure 4) indicates that the desired diameter (maximum value) can be achieved in two different compromise zones (red areas). The first zone lies along the axis of the triangle formed by the two essential oils, *E. camaldulensis* and *T. zygis*, with a minimization of the third essential oil, *L. stoechas*. The second compromise zone is along the axis of the triangle formed by *L. stoechas* and *T. zygis*, with a minimization of *E. camaldulensis*. These results are more apparent in the mixture and 3D traces, showing that the desired compromise zone is present in two mixing areas.

##### Study of Desirability

To identify the proportions of the three oils that yield the desired response, we utilized the desirability graph tool. This tool offers the optimal proportions with a level of compromise. The aim is to achieve the maximum inhibition zone values.

**Figure 5:**
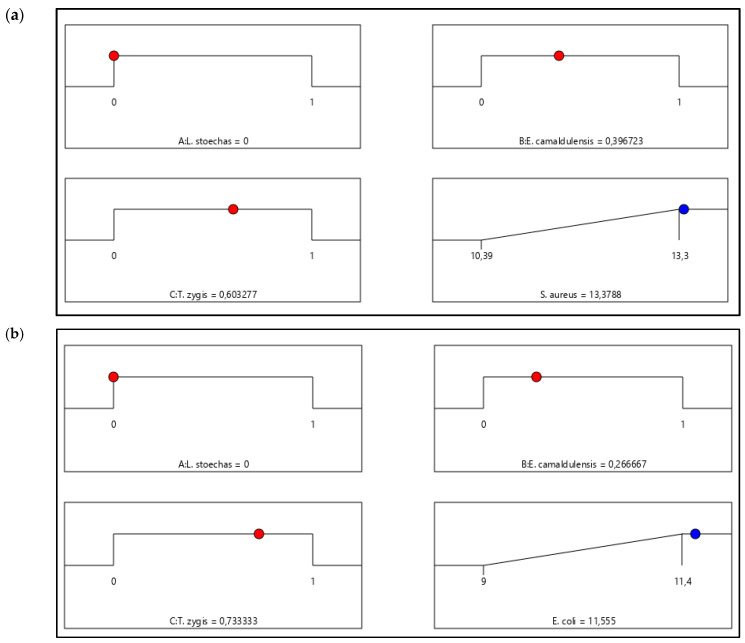

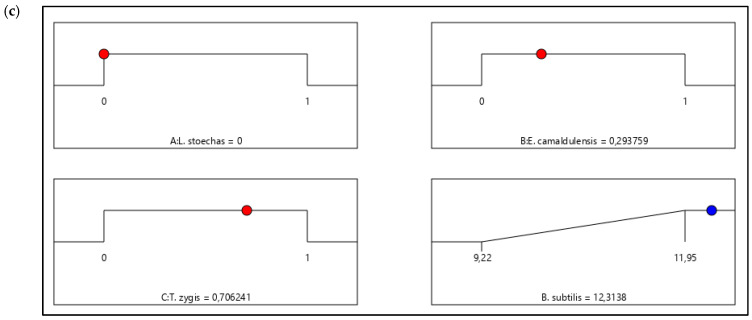
Desirability graph revealing the precise proportions of *T. zygis*, *L. stoechas* and *E. camaldulensis* EOs leading to the best antibacterial against *S. aureus* (**a**), *E. coli* (**b**) and *B. subtilis* (**c**).

The formulations below clearly indicate the proportions of individual EOs necessary to achieve the highest inhibition against the studied strains.

i. Effect of the Formulation against *S. aureus* ATCC: Figure 5a demonstrates that the maximum inhibition diameter achievable is 13.37 mm, with a desirability of 99.9%. According to the 2D and 3D mixture graph in Figure 4(a1), we can conclude that a mixture of *E. camaldulensis* EO and *T. zygis* EO is necessary to reach this inhibition zone value. Furthermore, this value can be achieved by making a mixture composed of 40% *E. camaldulensis* EO and 60% *T. zygis* EO.

ii. Effect of the Formulation against *E. coli* ATCC: Figure 5b illustrates that the maximum inhibition diameter attainable is 11.55 mm. additionally, the 2D and 3D mixture diagrams in Figure 4(a2) specify the exact proportions of *E. camaldulensis* EO and *T. zygis* EO required to achieve this value. The desirability test indicates a 99.9% probability of achieving the desired mixture with 27% *E. camaldulensis* EO and 73% *T. zygis* EO.

iii. Effect of the Formulation against *B. subtilis* ATCC: Figure 5c shows that the maximum inhibition diameter achievable is 12.31 mm, with a desirability of 99.9%. This value can be attained by creating a mixture of 29% *E. camaldulensis* EO and 71% *T. zygis* EO. Additionally, the 2D and 3D mixture diagrams in Figure 4(a3) indicate the precise proportions of *E. camaldulensis* EO and *T. zygis* EO needed to achieve this inhibition against *B. subtilis*.

Currently, many researchers employ mixture design methodology to analyze potential interactions between different components to identify optimal formulations [37,64,65]. To our knowledge, several studies have reported on the individual effects of selected essential oils, but no research has yet investigated the antibacterial capacity of combinations of *L. stoechas*, *T. zygis,* and *E. camaldulensis*. In this work, the augmented Simplex centroid model was used to optimize the antibacterial activity of essential oil mixtures against *B. subtilis*, *E. coli,* and *S. aureus*. This model reduces variability by examining multiple concentrations, ensuring a more comprehensive assessment of the antibacterial action of these EOs mixtures against these bacteria.

The results demonstrated that the binary mixture of 40% *E. camaldulensis* EO and 60% *T. zygis* EO is the necessary mixture to inhibit *S. aureus*. Also, the mixture of 27% *E. camaldulensis* EO and 73% *T. zygis* EO is the necessary mixture to achieve the optimal inhibitory concentration against *E. coli.* Additionally, the mixture of 29% *E. camaldulensis* EO and 71% *T. zygis* EO is the necessary mixture to inhibit *B. subtilis*. These findings suggest that combinations of thymus and eucalyptus essential oils in ratios of 0.40/0.60, 0.27/0.73, and 0.29/0.71 could be viable alternatives for food safety control against *S. aureus*, *E. coli*, and *B. subtilis*, respectively. In this context, Benkhaira et al. [66] concluded that the mixture of 36% *Ruta montana* and 64% *Clinopodium nepeta* is the optimal combination to limit the variability of *Staphylococcus aureus*. Recently, Jeddi et al. [67] showed that 32% *Eucalyptus polybractea cryptonifera*, 28% *Ormenis mixta*, and 40% *Lavandula burnatii briquet* comprise the optimal mixture against *Escherichia coli.* Meanwhile, 35% *Eucalyptus polybractea cryptonifera*, 30% *Ormenis mixta* and 35% *Lavandula burnatii briquet* make up the optimal mixture against *Staphylococcus aureus*. On the other hand, Chraibi et al. [9] reported that 60% and 40% for *Thymus satureioides* and *Myrtus communis* and 72% and 28% for *Thymus satureioides* and *Artemisia herba alba* predicted the highest antimicrobial effect against *Escherichia coli* and *Staphylococcus aureus*, respectively. However, the optimal mixture against *E. coli* and *S. aureus* corresponds to 54%/46% and 56%/44% of *Mentha pulegium* and *Mentha piperita* EOs, respectively [61].

The synergistic action of combined EOs could be due to the activity of their chemical compounds, particularly δ-terpineol, δ-3-carene, thymol, camphor, fenchone, 1,8-cineole, γ-terpinene, and α-terpineol, which are the main components of the studied essential oils. To our knowledge, the interactions between these molecules have not been previously documented. However, prior research has shown that combinations of 1,8-cineole/thymol, 1,8-cineole/limonene, α-pinene/linalool, and 1,8-cineole/ρ-cymene exhibit synergistic antibacterial activity [68,69,70]. Additionally, another study found that combinations of *Lavandula latifolia* and camphor were relatively more effective against the pathogens *Listeria monocytogenes* and *Staphylococcus aureus* [71].

In general, the antibacterial activity of essential oils is linked to the functional groups present in their active components, which can bind to the cell surface and penetrate the phospholipid bilayer of the cell membrane. This accumulation disrupts the membrane’s structural integrity, altering cellular metabolism and leading to cell death [9]. For instance, in bacteria, it has been shown that phenolic groups like thymol interact with the outer membrane’s constituents, causing degradation and the release of lipopolysaccharides (LPS), which increases membrane permeability and results in significant ATP loss [67]. Similarly, compounds such as α-terpineol, terpinol-4-ol, and δ-terpineol destroy the membrane and cell wall integrity, altering permeability and releasing intracellular substances like nucleic acids and proteins [5]. A study by [72] also demonstrated that 1,8-cineole induces significant outer membrane degradation, cytoplasm reduction, and alters the cell’s physical characteristics. Likewise, camphor disrupts the bacterial membrane’s integrity, leading to bacterial death [63,73].

The combination of 1,8-cineole, camphor, and thymol shows significant antibacterial effects, particularly by disrupting bacterial cell membranes, such as those of *S. aureus* and *E. coli.* When used together, these compounds increase membrane permeability, leading to the leakage of intracellular substances and compromising cell integrity. Previous studies have shown that the synergistic interactions between thymol and 1,8-cineole promote structural changes within the membranes, facilitating the influx of antimicrobial compounds and the release of essential ions, thereby disrupting bacterial metabolic functions [74,75]. However, the specific role of camphor in this combination remains to be explored. Research indicates that camphor, due to its terpenoid structure and lipophilic properties, could enhance the membranotropic effects of the other compounds, disrupting bacterial membrane integrity, which may increase membrane fluidity and permeability [76]. This potential synergy could involve cooperative action by modifying bacterial membrane fluidity, increasing permeability, and causing the leakage of vital components such as ions. This disruption leads to potential membrane collapse and bacterial death. Exploring this possibility could provide a deeper understanding of antibacterial mechanisms and contribute to the development of more effective therapeutic strategies.

Overall, the findings of this study provide scientific evidence supporting the potential applications of combined oils to develop new effective antimicrobial agents against resistant bacterial strains. These agents could be beneficial in food packaging and preservation, minimizing the loss of nutritional and organoleptic properties of various food products, as well as in the development of biopharmaceutical products [37,64]. Indeed, essential oils and their components offer solutions to combat bacteria that are safer and more environmentally friendly. Many bioactive molecules are selective and less toxic to humans, animals, and the environment.

## 4. Conclusions

The present study demonstrated that essential oils extracted from *L. stoechas*, *T. zygis,* and *E. camaldulensis* exhibit significant antimicrobial potential. Whether used alone or in combination, these volatile oils are highly effective against bacterial strains *S. aureus*, *E. coli,* and *B. subtilis*. The antibacterial capacity of the selected essential oils depends on their proportions in the formulation. According to the mixture design, the sensitivity of the bacterial strains can be attributed to the synergistic effect between the active constituents of the combined oils. The combination of *Tz/Ec* and *Ls/Ec/Tz* in the ratios 0.5:0.5 and 1/6:1/6:2/3, respectively, showed higher inhibition diameters against Gram-negative strains than against Gram-positive ones. This could be related to higher levels of δ-3-Carene, 1,8-cineole, camphor, and δ-terpineol and the synergistic effect between them.

The binary mixture of 40% *E. camaldulensis* EO and 60% *T. zygis* EO constitutes the optimal mixture for inhibiting *S. aureus*. Similarly, the mixture of 27% *E. camaldulensis* EO and 73% *T. zygis* EO is necessary to achieve the optimal inhibitory concentration against *E. coli*. Additionally, the mixture of 29% *E. camaldulensis* EO and 71% *T. zygis* EO is necessary to inhibit *B. subtilis*. These combinations can serve as alternatives to conventional antibiotics, whose effectiveness is diminishing against certain resistant strains that cause serious pathologies in medicine and quality degradation in the food industry. These results should be considered for the successful application of these natural preservatives in the food industry.

## Figures and Tables

**Table 1:** Experience matrix of augmented centroid design.

Experiment Number	*L. stoechas*	*E. camaldulensis*	*T. zygis*
1	1	0	0
2	0	1	0
3	0	0	1
4	0.5	0.5	0
5	0.5	0	0.5
6	0	0.5	0.5
7	1/3	1/3	1/3
8	1/3	1/3	1/3
9	1/3	1/3	1/3
10	2/3	1/6	1/6
11	1/6	2/3	1/6
12	1/6	1/6	2/3

**Table 2:** Chemical composition in percentage of the essential oils of *L. stoechas* (*Ls*), *T. zygis* (*Tz*) and *E. camaldulensis* (*Ec*) and their combinations analyzed by GC/MS.

No	Compounds	RI	*Ls*	*Tz*	*Ec*	*Ls/Tz* (0.5:0.5)	*Ls/Ec* (0.5:0.5)	*Tz/Ec* (0.5:0.5)	*Ls/Ec/Tz* (1/3:1/3:1/3)	*Ls/Ec/Tz* (2/3:1/6:1/6)	*Ls/Ec/Tz* (1/6:2/3:1/6)	*Ls/Ec/Tz* (1/6:1/6:2/3)
1	Heptanal	901	-	0.14	-	-	0.07	-	-	-	-	-
2	Santalina triene	906	0.34	-	-	-	0.11	-	-	0.17	0.05	0.19
3	Tricyclene	921		1.45	-	0.17	-	0.18	-	-	-	-
4	α-Thujane	924	4.33	0.05	3.49	2.31	4.45	2.59	3.35	3.79	2.73	2.45
5	α-Pinene	932	4.24	3.98	0.32	3.39	2.14	2.3	3.15	3.58	1.2	3.6
6	Norbornen-2-ol	941	-	0.11	-	-	-	-	-	-	-	-
7	Fenchene	946	0.21	-	-	-	0.09	-	0.1	0.14	-	-
8	Verbinene	961	0.12	1.65	-	0.86	0.09	1.51	1.27	0.81	1.21	1.23
9	Sabinene	969	0.28	0.13	0.25	-	1.02	-	-	-	-	-
10	β-Pinene	974	0.19	0.24	0.35	0.19	0.28	0.29	0.31	0.24	0.26	0.25
11	cis Pinane	982	0.14	-	-	0.17	-	-	0.12	0.12	-	0.14
12	Myrcene	988	-	0.07	-	0.09	-	-	-	-	-	-
13	oxide linalool	991	0.07	-	-	-	-	-	-	-	-	-
14	2-Octanol	994	-	-	0.1	-	0.23	0.27	0.22	0.18	0.3	-
15	δ-2-Carene	1001	2.28	0.37	0.1	0.93	1.26	0.32	0.79	1.49	0.33	0.39
16	δ-3-Carene	1008	-	15.7	0.06	8.87	-	11.31	8.74	5.06	6.22	12.27
17	α-Terpinene	1014	1.05	0.42	-	-	0.08	-	-	-	-	-
18	ρ-Cymene	1020	1.26	2.44	4.93	0.79	4.74	-	-	1.46	-	-
19	Limonene	1024	0.89	-	-	1.2	-	-	-	-	-	-
20	1,8-cineol	1026	-	-	43.61	-	24.53	21.93	14.32	8.39	28.77	9.76
21	E,β-Ocymene	1044	0.19	1.85	-	2.65	0.05	-	-	-	-	-
22	γ-Terpinene	1054	-	0.72	11.71	0.59	2.91	5.84	4.08	2.32	4.98	4.66
23	cis hydrate Sabinene	1065	0.4	-	0.13	-	0.18	-	-	0.19	0.1	0.05
24	trans Oxide linalool	1067	-	0.6	-	7.67	-	-	-	-	-	-
25	Camphelilone	1078	-	1.38	0.85	1.77	-	0.53	0.54	-	-	-
26	Fenchone	1084	16.57	-	-	-	8.16	-	5.52	9.78	3.09	3.02
27	Terpinolene	1086	-	0.48	2.3	-	-	1.59	1.28	1.66	1.57	1.39
28	Linalool	1095	2.86	0.16	-	0.29		0.1	1.6	-	-	-
29	Trans-Hydrate Sabinene	1098	-	-	0.27	-	-	-	-	-	-	-
30	α-Fenchocamphorone	1104	0.28	-	-	-	0.34	-	0.22	0.38	0.23	0.14
31	6-Camphenol	1111	-	0.23	-	-	-	0.23	-	-	-	0.26
32	endo-Fenchol	1114	1.34	-	-	0.36	0.42	-	0.33	0.4	0.34	-
33	trans Hydrate Pinene	1119	-	-	0.04	-	-	-	-	-	-	-
34	dehydro Linalool	1131	-	4.99	-	-	-	-	-	-	-	-
35	trans-β-dihydro Terpineol	1134	-	-	0.39	-	-	-	-	-	-	-
36	cis hydrate Pinene	1139	-	-	1.42	-	0.57	-	-	-	-	-
37	Camphor	1141	36.15	-	-	23.77	23.6	-	15.82	28.94	9.91	10.03
38	β-Oxide-Pinene	1154	3.21	-	-	0.4	1.81	-	0.3	0.15	-	-
39	δ-Terpineol	1162	-	27.64	1.16	16.8	2.39	16.15	11.29	7.12	7.05	19.48
40	Thujanol	1164	-	1.12	-	-	-	-	-	-	-	-
41	cis Oxyde Linalool	1170	2.05	0.93	-	1.68	-	-	0.86	-	3.02	0.86
42	Terpinene-4-ol	1174	1.36	-	3.91	1.06	2.55	2.45	2.12	1.75	3.32	1.88
43	iso-Verbanol	1176	-	0.31	-	-	-	-	-	0.8	-	-
44	ρ-Cymen-8-ol	1179	0.38	-	-	0.3	-	-	1.13	0.6	-	-
45	neo-Verbanol	1182	-	0.84	-	0.41	-	-	-	-	-	-
46	α-Terpineol	1186	-	-	10.58	-	2.82	5.47	1.98	1.18	4.63	1.78
47	ϒ-Terpineol	1199	0.6	-	0.22	-	0.39	-	-	0.38	0.52	0.39
48	Verbenone	1204	1.23	0.05		0.35	0.55	-	0.39	0.7	0.24	0.11
49	trans piperitol	1207	-	-	0.11	-	-	0.06	-	-	-	-
50	acetate Octenol	1208	0.34	-	-	-	0.13	-	-	-	-	-
51	acetate Octanol	1211	-	-	-	-	-	-	0.15	0.22	0.06	0.22
52	Formate Linalool	1214	0.12	-	-	-	0.05	-	-	0.1	-	-
53	trans Carveol	1215	-	2.14	-	1	-	1.11	0.79	0.44	0.44	-
54	acetate Endo-Fenchyl	1218	0.11	-	-	-	-	-	-	-	-	-
55	cis acetate hydrate Sabinene	1219	-	-	0.37	-	-	-	-	-	-	-
56	cis-Carveol	1226	-	1.77	-	0.6	-	1.19	0.76	0.36	-	0.95
57	Tetra hydro-acetate Linalool	1231	0.22	-	-	0.25	-	-	-	-	-	-
58	Pulegone	1233	-	-	1.02	-	0.57	-	-	0.16	0.98	-
59	Carvone	1239	0.14	-	0.23	-	0.07	0.15	0.02	0.07	0.12	-
60	trans acetate hydrate Sabinene	1253	-	-	0.13	-	0.05	-	0.02	-	0.06	-
61	Carvenone	1255	0.05	-	-	-	-	-	-	-	-	-
62	cis oxide Carvone	1259	0.07	-	-	-	0.08	-	-	-	-	-
63	iso-3-acetate Thujanol	1267	0.12	-	0.33	0.07	0.44	0.42	0.32	0.14	0.62	0.16
64	trans-Oxide Carvone	1273	0.35	4.13	0.6	3.95	-	4	2.64	1.67	1.75	4.56
65	neoiso-3-acetate Thujanol	1281	-	-	0.2	-	0.23	-	-	-	0.35	-
67	trans-acetate Oxide Linalool	1287	0.05	-	-	-	-	-	-	-	-	-
68	Thymol	1289	-	14.17	-	5.91	-	6.81	4.43	2.28	2.8	7.84
69	ρ-Cymen-7-ol	1290	-	-	1.35	-	0.27	-	-	-	-	-
70	trans acetate Verbenyl	1291	-	0.06	-	-	-	-	-	-	-	-
71	acetate dehydro Carveol	1306	0.3	-	-	0.12	0.14	-	0.15	0.18	-	-
72	Iso acetate Verbanol	1308	0.26	0.31	-	0.18	0.08	0.16	0.14	0.1	0.05	0.21
73	δ-Acetate Terpinyl	1316	-	-	0.08	-	-	-	-	-	-	-
74	neo-iso acetate Verbanol	1328	0.02	0.02	-	-	-	-	-	-	0.16	-
75	δ-Elemene	1335	-	-	0.95	-	1.06	1.03	0.7	0.38	1.63	0.36
76	acetate Verbanol	1340	0.02	0.03	-	0.06	-	-	-	-	-	-
77	α-acetate Terpinyl	1346	-	-	0.51	-	-	0.03	0.03	0.07	-	0.04
78	cis-acetate Carvyl	1365	-	-	0.04	-	-	0.03	0.04	-	-	
79	α-Copaene	1374	-	0.08	-	-	-	-	0.04	-	-	0.04
80	β-Elemen	1389	0.15	-	-	0.05	0.05	-	0.05	0.07	-	-
81	β-Longipinene	1400	-	0.07	-	-	-	-	0.04	-	-	-
82	Longifolene	1407	-	-	0.05	-	-	0.05	-	-	-	-
83	E-Caryophyllene	1417	-	2.75	0.35	1.42	-	1.55	1.07	0.51	0.59	1.88
84	Carvone hydrate	1422	-	-	0.26	-	-	-	-	-	-	-
85	4,8-β-epoxy-Caryophyllane	1423	0.1	-	-	-	-	-	-	-	-	-
86	γ-Elemene	1434	-	0.07	-	-	-	-	-	-	-	-
87	Aromadendrene	1439	-	-	0.24	-	0.06	0.05	0.07	-	0.09	-
88	α-Humulene	1452	-	0.12	0.23	0.05	-	0.1	0.07	-	-	0.08
89	Sesquisabinene	1457	-	0.17	-	0.14	-	0.12	0.13	0.12	-	0.13
90	9-epi-E-Caryophyllene	1464	0.13	-	0.09	-	0.1	-	-	-	0.11	-
91	10-epi-β-Acoradiene	1474	0.05	-	-	-	-	-	-	-	-	-
92	β-Thujaplicin	1475	-	0.16	-	-	-	-	-	-	-	0.09
93	γ-Muurolene	1478	-	-	0.25	-	-	-	0.1	-	0.09	-
94	Germacrene D	1484	0.4	-	-	0.1	0.12	-	0.16	0.17	0.16	0.07
95	β-Selinene	1489	-	0.39	-	0.11	-	0.09	0.13	-	-	0.1
96	δ-Selinene	1492	0.23	-	0.2	-	-	-	-	0.11	0.11	-
97	α-Selinene	1498	0.07	-	-	-	-	-	-	-	-	-
98	α-Muurolene	1500	-	-	0.38	-	-	-	-	-	-	-
99	β-Bisaboline	1505	-	0.13	-	-	-	0.04	0.04	-	-	0.05
100	γ-Cadinene	1513	-	0.27	0.07	-	-	0.09	-	-	-	0.19
101	7-epi-α-Selinene	1520	0.68	-	-	0.41	0.25	-	0.25	0.45	0.13	-
102	δ-Cadinene	1522	-	-	0.09	-	-	-	-	-	-	-
103	α-Cadinene	1537	0.39	0.03	-	-	0.15	0.15	-	0.2	0.07	0.05
104	α-Calacorene	1544	1.18	-	-	0.51	0.52	-	0.35	0.78	0.2	0.16
105	Elemol	1548	-	0.03	2.11	-	0.41	0.38	0.23		0.68	0.11
106	β-Calacorene	1564	0.42	-	0.22	0.08	0.2	-	0.1	0.14	0.17	0.05
107	Davanone B	1564	-	-	-	-	-	0.07	-	-	-	-
108	Caryophyllenyl Alcohol	1570	-	0.21	-	0.2	-	-	-	-	-	-
109	Germacrene D-4-ol	1574	-	-	0.07	-	0.05	-	-	-	-	-
110	trans hydrate sesquisabinene	1577	0.05	0.22	-	-	-	-	1.14	0.57	-	0.79
111	Oxide Caryophellene	1582	-	2.24	0.3	1.51	1.4	1.77	1.35	0.74	2.43	2.04
112	Davanone	1587	-	-	1.1	-	0.57	1.87	-	-	1.34	-
113	cis-β-Elemenone	1589	-	0.12	-	-	-	-	-	-	-	-
114	Viridiflorol	1592	0.22	-	-	-	-	-	-	-	-	-
115	Widdrol	1599	-	-	0.23	-	0.18	0.26	0.15	-	0.23	0.13
116	trans-β-Elemenone	1601	-	0.09	-	-	-	-	-	-	-	-
117	trans Isolongifolanone	1612	-	-	0.08	-	-	0.1	-	-	-	-
118	Z-8-hydroxy Linalool	1619	8.28	-	-	3.83	4.02	-	2.76	5.1	1.86	1.38
119	trans Isolongifolanone	1625	-	-	0.28	-	0.17	-	0.04	-	0.1	0.02
120	E-Sesquilavandulol	1631	0.21	-	-	0.15	-	-	-	0.23	-	-
121	α-Acorenol	1632	-	-	0.15	-	-	-	-	-	-	-
122	cis-Cadin-4-en-7-ol	1635	-	0.69	-	0.62	-	0.68	0.53	0.61	0.52	0.55
123	epi-α-Cadinol	1638	0.59	-	-	-	0.46	-	-	-	-	-
124	epi-α-Muurolol	1644	-	-	0.18	-	-	-	-	-	-	-
125	β-Eudesmol	1649	-	0.35	-	-	-	-	-	0.73	-	0.13
126	α-Eudesmol	1652	-	0.28	-	-	-	0.28	-	-	-	-
127	1,2-dihydro-8-hydroxy-2E-Linalool	1654	0.63	-	-	0.33	0.53	-	0.55	-	-	-
128	dehydro-Eudesmol	1661	-	-	0.54	-	-	0.26	-	-	0.57	-
129	14-hydroxy-Z-Caryophyllene	1666	-	0.39	-	0.36	-	0.29	0.31	-	0.28	0.31
130	epi-β-Bisabolol	1670	0.39	-	-	-	0.07	-	-	0.4	-	-
131	acetate Davanol	1689	-	0.09	-	-	-	-	-	-	-	-
132	acetate caryophyllene	1701	0.7	-	-	0.32	0.34	-	0.23	0.44	0.14	0.15
133	2E,6Z-Farnesol	1714	0.23	-	0.1	-	0.8	0.06	0.05	0.1	-	-
134	2E,6Z Farnesol	1715		-	-	-	-	-	-	-	-	-
135	Z-acetate Sesquilavandyl	1732	0.06	-	-	-	0.06	-	-	-	-	-
136	2E,6E-Farnesol	1742	0.02	-	-	-	-	-	0.05	-	-	-
137	β-Acoradienol	1762	0.08	-	-	-	-	-	0.03	0.05	-	-
138	γ-acetate Eudesmol	1783	0.04	-	-	-	-	-	-	-	-	-
	Total		99.49	99.63	99.68	99.4	99.51	96.31	100.04	99.36	98.96	94.34
	Monoterpenes		84.19	94.1	90.61	89.15	87.94	86.96	89.25	87.5	87.46	88.27
	Sesquiterpenes		15.3	5.53	9.07	10.25	11.57	9.35	10.79	11.86	11.5	6.07
	Hydrocarbons		23.68	36.59	29.29	27.67	24.08	31.26	29.84	25.75	24.91	30.24
	Ketones		54.84	5.77	4.42	29.84	34.11	6.72	25.19	41.7	17.76	17.88
	Aldehydes		-	0.14	-	-	0.07	-	-	-	-	-
	Esters		0.17	0.06	0.63	-	0.06	0.06	0.07	0.07	-	0.04
	Ethers		-	-	43.61	-	24.53	21.93	14.32	8.39	28.77	9.76
	Alcohols		20.8	37.45	21.62	33.97	16.66	26.94	24.64	19.57	24.28	27.28
	Phenols		-	19.73	0.11	7.92	-	9.4	5.98	3.88	3.24	9.14

No: In order of elution on HP-5ms; Components: Components identified based on retention indices and mass spectra; RI: Retention indices calculated experimentally using homologous series of C9–C28 alkanes; -: Not detected.

**Table 3:** Antimicrobial activities of the essential oils against different strains of bacteria by disk diffusion method.

	Microorganisms	DI (mm)		
Plants		*Escherichia coli* (ATCC 8739)	*Bacillus subtilis* (ATCC 6633)	*Staphylococcus aureus* (ATCC 6538)
*T. zygis* (5 µL/disk)		16.11 ± 0.96	17.11 ± 0.30	18.33 ± 0.89
*E. camaldulensis* (5 µL/disk)		19.11 ± 0.30	22.56 ± 0.59	25.00 ± 0.22
*L. stoechas* (5 µL/disk)		15.78 ± 0.59	20.33 ± 0.44	20.78 ± 0.74
Tetracycline		24 ± 0.33	21.67 ± 0.44	25.5 ± 0.33

DI: diameter of inhibition; Tetracycline: antibiotic.

**Table 4:** Different combinations generated by the chosen mixture design and experimental responses.

Experiment Number	*L. stoechas*	*E. camaldulensis*	*T. zygis*	DI *S. aureus* (mm)	DI *E. coli* (mm)	DI *B. subtilis* (mm)
1	1	0	0	11.00 ± 0.74	10.44 ± 0.37	11.45 ± 0.59
2	0	1	0	10.39 ± 0.67	9.00 ± 0.74	9.22 ± 0.59
3	0	0	1	12.11 ± 0.19	11.22 ± 0.44	11.75 ± 0.81
4	0.5	0.5	0	11.64 ± 0.44	9.33 ± 0.22	11.67 ± 0.81
5	0.5	0	0.5	11.62 ± 0.37	11.33 ± 0.44	11.56 ± 0.44
6	0	0.5	0.5	13.30 ± 0.89	11.23 ± 0.59	11.89 ± 0.96
7	1/3	1/3	1/3	11.44 ± 0.59	10.67 ± 0.89	10.50 ± 0.44
8	1/3	1/3	1/3	11.33 ± 0.22	10.46 ± 0.52	10.30 ± 0.59
9	1/3	1/3	1/3	11.42 ± 0.15	10.56 ± 0.15	10.12 ± 0.30
10	2/3	1/6	1/6	10.89 ± 0.81	10.18 ± 0.37	11.34 ± 0.15
11	1/6	2/3	1/6	11.46 ± 0.22	9.98 ± 0.30	10.46 ± 0.96
12	1/6	1/6	2/3	11.97 ± 0.81	11.40 ± 0.44	11.95 ± 0.52

**Table 5:** Analysis of variance for the fitted model.

Source		Degrees of Freedom	Sum of Squares	Squares Medium	F Report	*p*-Value
*DI S. aureus*	Regression	6	5.64	0.9394	47.86	0.0003
	Residual	5	0.0981	0.0196		
	Total	11	5.73			
	Lack of fit	3	0.0913	0.0304	8.86	0.1031
	Pure error	2	0.0069	0.0034		
	*R* ^2^	0.9829				
	*R_adj_* ^2^	0.9624				
*DI E. coli*	Regression	6	6.50	1.08	69.30	0.0001
	Residual	5	0.0781	0.0156		
	Total	11	6.58			
	Lack of fit	3	0.0561	0.0187	1.69	0.3921
	Pure error	2	0.0221	0.0110		
	*R* ^2^	0.9881				
	*R_adj_* ^2^	0.9739				
*DI B. subtilis*	Regression	6	7.70	1.28	10.72	0.0099
	Residual	5	0.5984	0.1197		
	Total	11	8.29			
	Lack of fit	3	0.5261	0.1754	4.85	0.1756
	Pure error	2	0.0723	0.0361		
	*R* ^2^	0.9278				
	*R_adj_* ^2^	0.8413				

**Table 6:** Estimated regression coefficients for the fitted model.

Terme		Coefficient	Estimation	Std Error	t-Student	*p*-Value
DI *S. aureus*	*L. stoechas*	b1	10.94	0.1353	80.88	<0.0001 *
	*E. camaldulensis*	b2	10.39	0.1353	76.81	<0.0001 *
	*T. zygis*	b3	12.10	0.1353	89.40	<0.0001 *
	*L. stoechas* * *E. camaldulensis*	b12	3.67	0.6813	5.39	0.0030 *
	*L. stoechas* * *T. zygis*	b13	0.1237	0.6813	0.18	0.8631
	*E. camaldulensis* * *T. zygis*	b23	8.18	0.6813	12.01	<0.0001 *
	*L. stoechas* *E. camaldulensis * *T. zygis*	b123	−30.90	3.71	−8.34	0.0004 *
DI *E. coli*	*L. stoechas*	b1	10.38	0.1207	85.98	<0.0001 *
	*E. camaldulensis*	b2	9.03	0.1207	74.76	<0.0001 *
	*T. zygis*	b3	11.25	0.1207	93.18	<0.0001 *
	*L. stoechas* * *E. camaldulensis*	b12	−1.63	0.6080	−2.67	0.0441 *
	*L. stoechas* * *T. zygis*	b13	1.94	0.6080	3.19	0.0242 *
	*E. camaldulensis* * *T. zygis*	b23	4.59	0.6080	7.55	0.0006 *
	*L. stoechas* * *E. camaldulensis* * *T. zygis*	b123	−5.51	3.31	−1.67	0.1567
DI *B. subtilis*	*L. stoechas*	b1	11.47	0.3341	34.33	<0.0001 *
	*E. camaldulensis*	b2	9.18	0.3341	27.49	<0.0001 *
	*T. zygis*	b3	11.90	0.3341	35.62	<0.0001 *
	*L. stoechas* * *E. camaldulensis*	b12	5.34	1.68	3.18	0.0247 *
	*L. stoechas* * *T. zygis*	b13	0.2153	1.68	0.13	0.9031
	*E. camaldulensis* * *T. zygis*	b23	5.84	1.68	3.47	0.0178 *
	*L. stoechas* * *E. camaldulensis* * *T. zygis*	b123	−45.09	9.15	−4.93	0.0044 *

* Statistically significant.

## Data Availability

All related data are contained within the manuscript.
